# Association between preoperative albumin levels and postoperative delirium in geriatric hip fracture patients

**DOI:** 10.3389/fmed.2024.1344904

**Published:** 2024-02-14

**Authors:** Wei Wang, Wei Yao, Wanyun Tang, Yuhao Li, Qiaomei Lv, Wenbo Ding

**Affiliations:** ^1^Department of Orthopedics, Dandong Central Hospital, China Medical University, Dandong, China; ^2^Department of Oncology, Dandong Central Hospital, China Medical University, Dandong, China

**Keywords:** hip fracture, albumin levels, hypoalbuminemia, postoperative delirium, POD

## Abstract

**Objective:**

This study aims to examine the association between preoperative serum albumin levels and postoperative delirium (POD) in geriatric patients who have undergone hip fracture surgery, with the goal of offering novel insights for clinical interventions targeting POD.

**Methods:**

A retrospective analysis was conducted on the medical records of patients who underwent hip fracture surgery in a tertiary medical institution from January 2013 to November 2023. The patients were classified based on hypoalbuminemia (defined as a serum albumin level < 35 g/L) and clinical threshold. Multivariable logistic regression and propensity score matching analysis (PSM) were employed to calculate the adjusted odds ratios (OR) and 95% confidence intervals (95% CI) for POD to eliminate potential confounding factors. Additionally, subgroup analysis was performed to explore the interaction effect.

**Results:**

The retrospective cohort study included 1,440 patients, with an incidence of POD found to be 19.1%. In a multivariable logistic regression analysis, patients with hypoalbuminemia had an adjusted OR of 2.99 (95%CI: 2.14–4.18) compared to those with normal albumin levels (≥ 35 g/L). Furthermore, a significant trend was observed across different severity categories, including mild hypoalbuminemia (34.9–30.0 g/L; adjusted OR = 2.71, 95%CI: 1.84–3.99), moderate hypoalbuminemia (29.9–25.0 g/L, adjusted OR = 3.44, 95%CI: 1.88–6.28), and severe hypoalbuminemia (<25.0 g/L; adjusted OR = 3.97, 95%CI: 1.78–8.86), with a trend value of *p* <0.001. Similar results were observed in the PSM analysis. Additionally, treating preoperative serum albumin level as a continuous variable, the risk of POD increased by 11% (95% CI, 1.08–1.15) with each 1 g/L decrease in preoperative serum albumin level.

**Conclusion:**

Low preoperative levels of albumin are strongly associated with POD in geriatric patients with hip fractures, and a significant dose–response relationship exists between them.

## Introduction

Due to the progressive aging of the global population, it is anticipated that the number of hip fractures worldwide will increase to an estimated 6.1 million cases by 2050, with an annual escalation of 1 to 3% (1). Postoperative delirium (POD) is a frequent and severe complication in geriatric patients who have undergone hip fracture surgery, with an incidence rate ranging from 4 to 53% (2–10). POD is an acute cognitive dysfunction characterized by changes in consciousness or attention that cannot be attributed to pre-existing cognitive impairment (2, 8, 9). Hallucinations and delusions, restlessness, disturbed speech, decreased attention, disordered thinking, agitated behavior, and disturbances in sleep–wake cycles are the most prevalent symptoms (5, 7). POD usually occurs within the first 5 days after surgery, predominantly within the first 48 h (7). Numerous studies have demonstrated that POD has a significant impact on surgical outcomes, cognitive recovery, and healthcare costs (2). Furthermore, POD is associated with prolonged hospital stays, increased mortality rates at 6 months and 1 year, and the development of permanent cognitive impairment (5, 11).

Multiple prospective studies have demonstrated that hypoalbuminemia can contribute to the occurrence of POD. In one such study, Venkatakrishnaiah et al. (10) prospectively evaluated 110 geriatric hip fracture patients and found that hypoalbuminemia was a strong predictor of POD. Similarly, Ishihara et al. (12) conducted a multicenter prospective study and showed that serum albumin concentration ≤ 37 g/L was an independent risk factor for POD in patients undergoing initial hepatectomy. Zhang et al. (13) investigated preoperative hypoalbuminemia in a clinical trial involving geriatric ICU patients after surgery and reported an increased risk of POD and poor prognosis. Finally, a multicenter prospective study conducted by Matsuki et al. (14) revealed a correlation between low serum albumin levels and the incidence of POD in urological surgery patients.

While several studies (2, 5–10, 15) have confirmed the relationship between preoperative serum albumin levels and POD, the general and oversimplified conclusions have limited the specificity of clinical practices and hindered the promotion of preoperative albumin management. Currently, focusing on modifiable risk factors for intervention represents the most effective approach to prevent adverse outcomes of POD (6, 16). This study aims to employ a retrospective cohort design to meticulously classify preoperative serum albumin levels and investigate the strength of the association between different levels of preoperative serum albumin and POD. The objective is to provide clinicians with precise and quantitative predictive associations and determine the threshold level of preoperative albumin that benefits patients. This research will aid in enhancing the feasibility of clinical interventions and offer new evidence for the routine assessment and management of preoperative albumin levels in hip fracture patients.

This study seeks to address several critical questions through its retrospective cohort design:

1. Emphasize modifiable and clinically manageable risk factors to provide novel insights into interventions for POD in hip fracture patients.
2. Quantify and visually represent the intensity of risk for POD associated with different preoperative serum albumin levels, thereby enhancing the clinical feasibility and practical value of previous research findings.
3. Determine the dose–response relationship between preoperative serum albumin levels and POD, while examining potential interactions with other risk factors.

## Methods

### Study design and data collection

The present study was a retrospective cohort investigation that collected electronic medical records from our institution between January 2013 and November 2023. The data collection process was independently conducted by two authors (WW and WY), and any discrepancies were rechecked to ensure accuracy. This study followed the ethical principles of the 1964 Helsinki Declaration and received approval from the Institutional Review Board (IRB) for all aspects of the study, waiving the requirement for written informed consent.

### Patient selection

**Figure 1:**
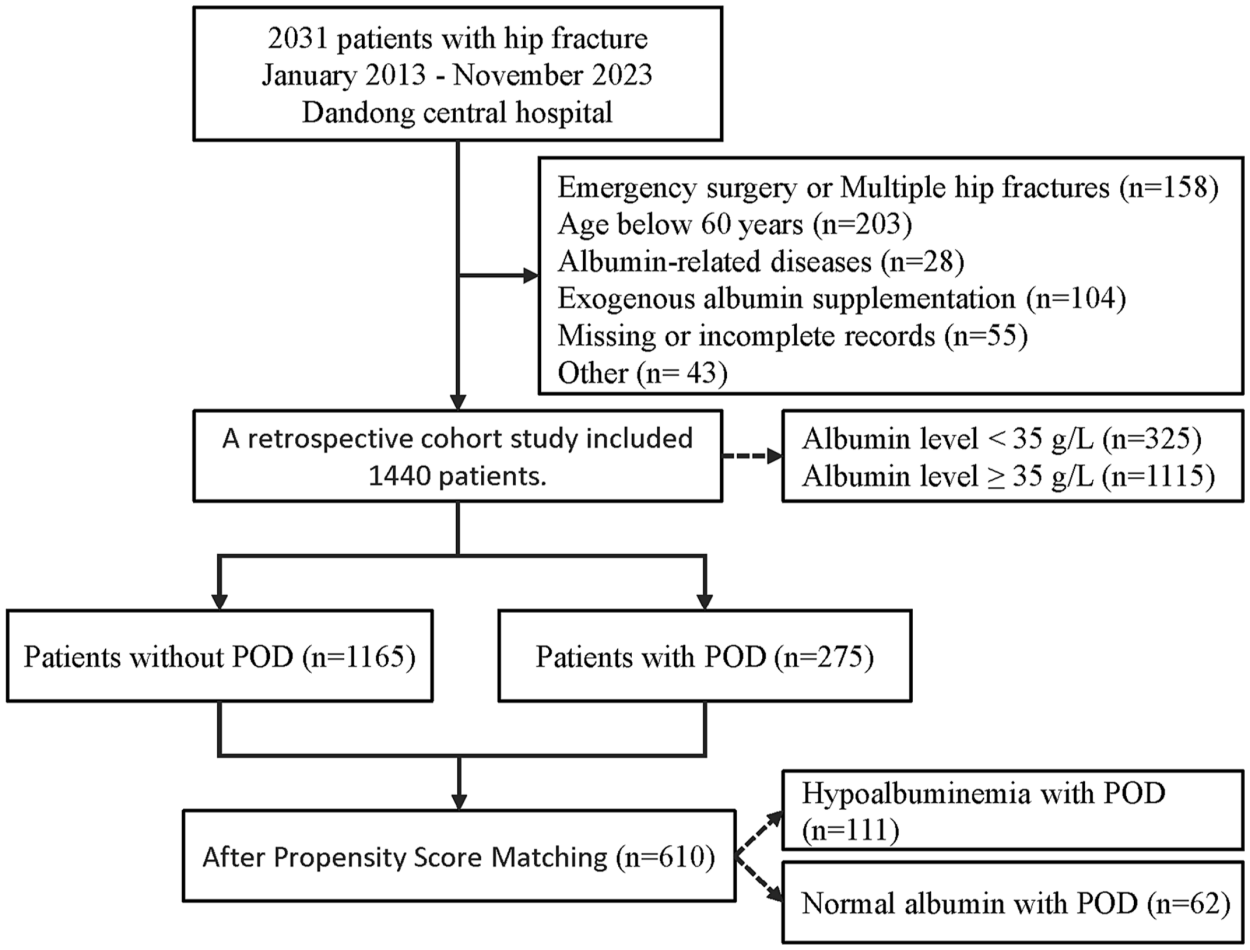
Flow diagram for selection of cohorts.

The study included patients with hip fractures who underwent surgical treatment, but excluded patients who met any of the following criteria: (1) multiple or pathological hip fractures; (2) emergency or urgent surgery; (3) age under 60; (4) no laboratory tests, such as serum albumin, collected within 48 h before surgery, or incomplete or inaccessible electronic medical records; (5) factors directly interfering with albumin levels, such as exogenous albumin supplementation, nephritis, cirrhosis, and hematological diseases (leukemia, lymphoma); (6) neurological and psychiatric disorders other than dementia. The specific screening process is presented in Figure 1.

### Exposure

In our hospital, routine laboratory tests such as complete blood count and biochemistry were performed on patients within 48 h before surgery, and blood samples were collected, processed and analyzed according to the routine protocols of international biochemical laboratories. Hypoalbuminemia was defined as serum albumin level < 35 g/L, in accordance with the guidelines of the American Association for Clinical Chemistry (AACC) and the European Federation of Clinical Chemistry and Laboratory Medicine (EFLM). Mild, moderate, and severe hypoalbuminemia were defined as serum albumin levels of 34.9–30.0 g/L, 29.9–25.0 g/L, and < 25.0 g/L, respectively. Based on these albumin level categories, a dose–response linear relationship was explored.

### Outcome

POD was extracted from the daily medical records of attending physicians and postoperative visits by anesthesiologists. Anesthesiologists assessed patients’ consciousness at the bedside every 8 h within 24 h after surgery. Experienced psychiatrists were consulted for bedside diagnosis if patients exhibited symptoms similar to POD. The attending physicians recorded the diagnosis results and treatment recommendations. A diagnosis of delirium followed the criteria established by the American Psychiatric Association in the Diagnostic and Statistical Manual of Mental Disorders, Fifth Edition (DSM-5, 2013), usually utilizing the Confusion Assessment Method (CAM) as a diagnostic tool (17), which includes the following criteria: (1) acute onset, fluctuating course; (2) inattention; (3) disorganized thinking; (4) altered level of consciousness. A diagnosis of delirium required patients to present with both item 1 and item 2, along with item 3 or item 4 (8, 11, 15). It should be noted that in our hospital, patients who undergo surgical treatment for traumatic hip fractures receive supervised rehabilitation exercises from professional physicians. As such, the patient cohort included in this study has an adequately extended hospital stay to enable observation and recording of outcomes.

### Covariates

This study collected variables from medical records based on previously identified risk factors for POD (see Supplementary Table 1) and divided them into four groups: demographic, comorbidities, surgery-related factors, and preoperative laboratory tests. Collinearity tests were conducted to determine the final covariates included in the analysis. The variance inflation factor (VIF) was calculated for each variable to assess the presence of collinearity, with a VIF value exceeding 10 indicating a strong collinear relationship with other variables. Given that this study primarily involved geriatric patients, the inclusion of the frailty index (FI) variable, constructed using 27 items (14 diseases, 10 symptoms or signs, and 3 physical measurement indicators), was useful in comprehensively assessing their physical condition and increasing the value of the study. The FI is calculated by dividing the number of diseases or deficits an individual meets by the total number of included diseases or deficits (i.e., 28), ranging from 0 to 1. A higher value of the FI indicates a greater degree of frailty in an individual. We defined individuals as robust when FI ≤ 0.1, prefrail when FI > 0.1 and < 0.25, and frail when FI ≥ 0.25. Please refer to Supplementary Table 2 for more details.

The detailed covariates included in the analysis are as follows: demographic variables such as age, gender, body mass index (BMI), smoking status, and frailty index. Comorbidities included dementia, diabetes, heart failure, and preoperative delirium. Surgery-related factors included the American Society of Anesthesiologists (ASA) classification, time to surgery, duration of surgery, intraoperative blood loss, and transfusion. Preoperative laboratory tests included neutrophil count, lymphocyte count, and blood glucose levels. It should be noted that all patients with traumatic hip fractures in our hospital received general anesthesia, resulting in no differences in the collected medical records regarding anesthesia type.

### Statistical analysis

Since the continuous data did not meet the assumption of normality as determined by the Kolmogorov–Smirnov test, the baseline characteristics of patients were represented using the median (interquartile range) or numbers (percentages). To determine if there was a statistically significant trend in variables across multiple ordered albumin groups, the Jonckheere-Terpstra test was employed. To address potential statistical errors arising from multiple comparisons, Bonferroni correction was applied. Multiple logistic regression analysis was used to calculate adjusted odds ratios (ORs) and 95% confidence intervals (CIs), with covariates showing statistical significance in univariate logistic regression analysis included in further multivariate logistic regression models.

To minimize bias interference, we performed propensity score matching (PSM) on all included covariates. In the PSM 1:1 matching, we applied a nearest neighbor matching algorithm to match the hypoalbuminemia group with the normal albuminemia group, with a caliper value of 0.1 standard deviation. We used absolute standardized mean difference (SMD) to detect any imbalance between the two groups, considering SMD ≥ 0.10 as an indication of imbalance. Post-matched data from PSM were then used for logistic regression analysis to obtain PSM-adjusted ORs and 95% CIs.

Subgroup analysis was used to further explore the interaction between variables. Variables that exhibited synergistic effects on preoperative albumin levels were identified in order to address the challenge of correcting POD through the modification of a single risk factor alone. Subgroups were formed based on covariates, and each subgroup underwent independent univariate logistic regression analysis. By comparing the differences in ORs between various subgroups, we evaluated the presence of interaction.

Statistical significance was defined as a two-sided value of p less than 0.05. All statistical procedures were performed using SPSS Statistics 25.0 for Windows (IBM Corp., Armonk, NY) and R software version 4.3.1 for Windows (R Foundation for Statistical Computing, Boston, MA, United States).

## Results

From January 2013 to November 2023, a total of 2031 electronic medical records were collected. After applying strict inclusion and exclusion criteria, a retrospective cohort study was conducted on 1,440 patients. Among them, 325 patients (22.6%) had serum albumin levels below 35 g/L, and 275 patients (19.1%) experienced POD. The study population had an average hospital stay of 12 days. The selection process is illustrated in Figure 1.

**Figure 2:**
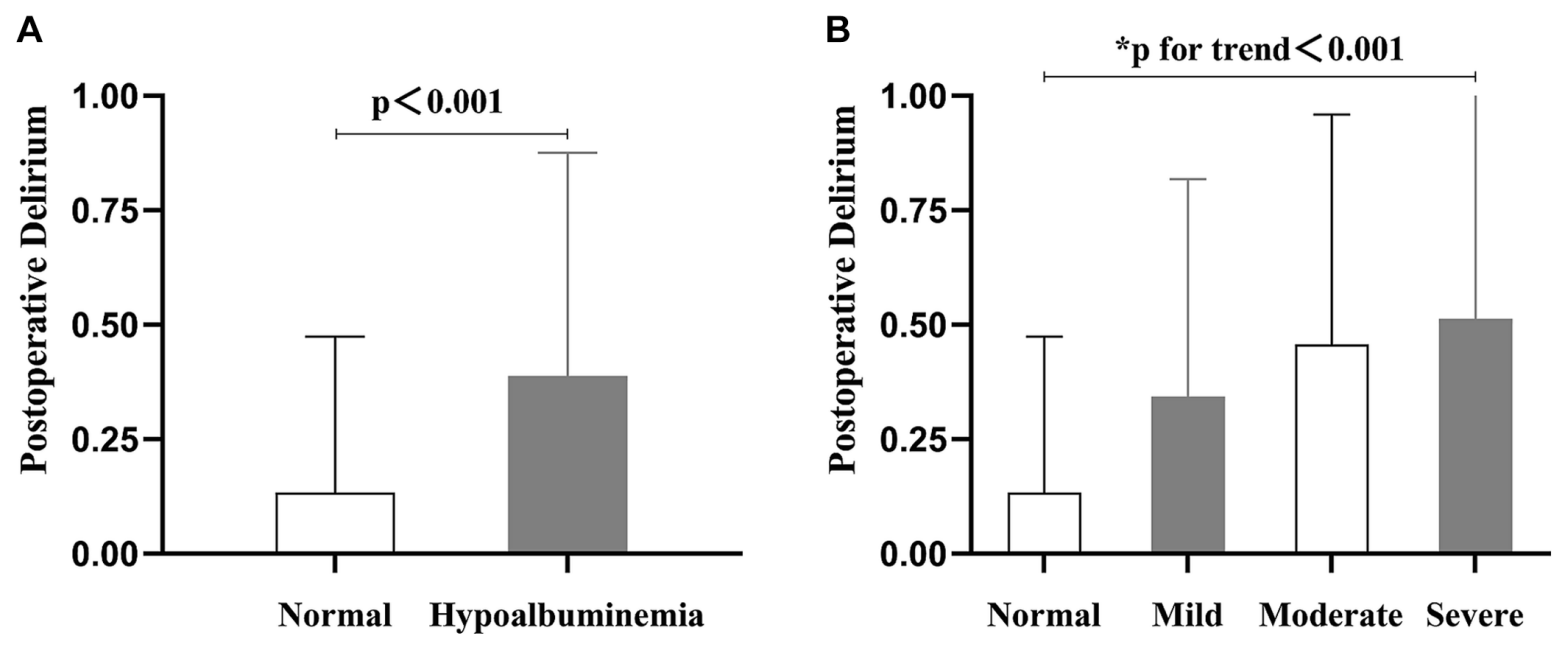
The column chart presents the incidence of postoperative delirium in relation to varying preoperative serum albumin levels. **(A)** The incidence of postoperative delirium was 13.4% in the group with normal preoperative serum albumin levels, and 38.8% in the hypoalbuminemia group. **(B)** The incidence of postoperative delirium varied across different serum albumin levels: 13.4% in the normal level group, 34.3% in the mild hypoalbuminemia group, 45.7% in the moderate hypoalbuminemia group, and 51.3% in the severe hypoalbuminemia group.

Table 1 presents the basic characteristics of the patients, categorized according to the severity of preoperative hypoalbuminemia. The distribution of patients in the normal albumin level group, mild, moderate, and severe hypoalbuminemia groups was as follows: 1115 patients (77.4%), 216 patients (15.0%), 70 patients (4.9%), and 39 patients (2.7%), respectively. The average age of all included patients was 75 years, with males accounting for 39.7%. Among the patients, 6.0% had dementia, 24.5% had diabetes mellitus. The Jonckheere-Terpstra test demonstrated a statistically significant trend in various variables as serum albumin levels increased. These variables include age, Frailty index, dementia, preoperative delirium, ASA classification, time to surgery, intraoperative blood loss, transfusion, lymphocyte count, and blood glucose level. Moreover, please consult Table 1 for the revised results obtained through the application of the Bonferroni method. Additionally, compared to the normal albumin level group, the hypoalbuminemia group had a higher incidence of POD (Figures 2A, *p* < 0.001), and the incidence of POD increased with the severity of hypoalbuminemia (Figure 2B; trend *p* < 0.001).

**Table 1:** Baseline characteristics of the patients based on preoperative albumin levels (g/L).

Characteristics	Total patients (*n* = 1,440)	Clinical classification of albumin levels (g/L)				P for Trend
		Normal albumin level (≥35.0, *n* = 1,115)	Mild hypoalbuminemia (34.9–30.0, *n* = 216)	Moderate hypoalbuminemia (29.9–25.0, *n* = 70)	Severe hypoalbuminemia (<25.0, *n* = 39)	
Demographics						
Age, years (Median, IQR)	75 (16)	74 (16)^a b^	80 (14) ^a^	83 (12) ^b c^	75 (16) ^c^	<0.001
Male (n, %)	571(39.7%)	437(39.2%)	89(41.2%)	27(38.6%)	18(46.2%)	0.499
BMI ≥30.0 kg/m^2^ (n, %)	293(20.4%)	239(21.4%)	29(13.4%)	9(12.9%)	16(41.0%)	0.119
Smoking (n, %)	242(16.8%)	193(17.3%)	25(11.6%)	8(11.4%)	16(41.0%)	0.575
Frailty index (FI)						
Robust (FI ≤ 0.1)	769(53.4%)	664(59.6%) ^a b c^	78(36.1%) ^a^	16(22.9%) ^b^	11(28.2%) ^c^	<0.001
Prefrail (FI > 0.1 and < 0.25)	574(39.9%)	391(35.1%)	119(55.1%)	47(67.1%)	17(43.6%)	
Frail (FI ≥ 0.25)	97(6.7%)	60(5.4%)	19(8.8%)	7(10%)	11(28.2%)	
Comorbidities						
Dementia (n, %)	86(6.0%)	51(4.6%) ^a b^	21(9.7%) ^a^	11(15.7%) ^b^	3(7.7%)	<0.001
Diabetes (n, %)	353(24.5%)	270(24.2%)	48(22.2%)	15(21.4%)	20(51.3%)	0.423
Heart Failure (n, %)	239(16.6%)	178(16%)	39(18.1%)	21(30.0%)	1(2.6%)	0.247
Preoperative delirium (n, %)	150(10.4%)	93(8.3%) ^a^	30(13.9%) ^b^	10(14.3%) ^c^	17(43.6%) ^a b c^	<0.001
Operative-related factors						
ASA classes ≥III (n, %)	814(56.5%)	593(53.2%) ^a^	154(71.3%) ^a^	46(65.7%)	21(53.8%)	<0.001
Time to surgery, days (Median, IQR)	5 (3)	5 (4) ^a^	6 (4) ^a^	5 (4)	5 (2)	0.01
Duration of surgery, hours (Median, IQR)	1.50 (0.80)	1.43 (0.75)	1.50 (0.85)	1.54 (0.70)	1.50 (0.50)	0.07
Operative blood loss, ml (Median, IQR)	120 (110)	120 (116)	150 (100)	193.5 (116)	127 (20)	0.008
Blood transfusion (n, %)	243(16.9%)	165(14.8%) ^a^	44(20.4%) ^b^	18(25.7%)	16(41.0%) ^a b^	<0.001
Preoperative laboratory tests						
NEU count, ×10^9^/L (Median, IQR)	6.40 (3.30)	6.50 (3.40)	6.27 (3.88)	5.80 (2.47)	6.50 (1.70)	0.203
LYM count, ×10^9^/L (Median, IQR)	1.20 (0.70)	1.23 (0.74) ^a b^	1.10 (0.70) ^a^	1.20 (0.77)	1.02(0.17) ^b^	<0.001
Blood glucose, mmol/L (Median, IQR)	6.20 (2.20)	6.10 (2.10) ^a^	6.20 (1.97) ^b^	6.30 (2.65) ^c^	8.30 (1.60) ^a b c^	0.009

^a–d^Statistically significant differences were observed between groups marked with the same superscript (Bonferroni correction). IQR, Interquartile Range; BMI, Body Mass Index; ASA, American Society of Anesthesiologists; NEU, Neutrophils; LYM, Lymphocyte.

Multiple logistic regression analysis, adjusted for covariates (see Supplementary Table 3 for details), was conducted, and the results are presented in Table 2. When the preoperative albumin level was treated as a continuous variable, the association between preoperative albumin level and POD was demonstrated with an odds ratio (OR) of 1.11 (95% CI: 1.08–1.15, *p* < 0.001). In other words, for every 1 g/L decrease in albumin level, the risk of POD increased by 11%. When categorized into two groups, the hypoalbuminemia group had an OR of 2.99 (95% CI: 2.14–4.18, *p* < 0.001) compared to the normal albumin level group. According to the severity of hypoalbuminemia, the adjusted ORs were 2.71 (95% CI: 1.84–3.99) for mild hypoalbuminemia, 3.44 (95% CI: 1.88–6.28) for moderate hypoalbuminemia, and 3.97 (95% CI: 1.78–8.86) for severe hypoalbuminemia, with a trend *p* < 0.001.

**Table 2:** Unadjusted and adjusted association between preoperative albumin levels and postoperative delirium.

	Albumin (g/L)	Events, n (%)	Unadjusted OR (95% CI)	*p*	Multivariable regression adjusted OR (95% CI)	*p*	PSM adjusted OR (95% CI)	*p*
Continuous	Per 1	NA	1.15 (1.12–1.19)	<0.001	1.11 (1.08–1.15)	<0.001	NA	NA
Dichotomy	Normal, ≥ 35	149(54.2%)	1 [Reference]	<0.001	1 [Reference]	<0.001	1 [Reference]	<0.001
	HypoAlb, < 35	126(45.8%)	4.11 (3.10–5.44)		2.99 (2.14–4.18)		2.24 (1.56–3.23)	
Clinical threshold	Normal, ≥ 35	149(54.2%)	1 [Reference]	<0.001*	1 [Reference]	<0.001*	1 [Reference]	<0.001*
	Mild, 34.9–30	74(26.9%)	3.38 (2.43–4.69)		2.71 (1.84–3.99)		2.09 (1.34–3.25)	
	Moderate, 29.9–25.0	32(11.6%)	5.46(3.31–9.01)		3.44 (1.88–6.28)		1.70 (0.83–3.47)	
	Severe, <25.0	20(7.3%)	6.82(3.56–13.09)		3.97 (1.78–8.86)		3.07 (1.05–8.93)	

HypoAlb, Hypoalbuminemia; NA, Not Applicable; CI, Confidence Interval; OR, Odds Ratio; PSM, Propensity Scores Matching. *p for trend.

Furthermore, 1:1 propensity score matching was performed between the normal albumin level group and the hypoalbuminemia group to balance all covariates (SMD < 0.1). The baseline characteristics of patients before and after matching are displayed in Supplementary Table 4. Logistic regression analysis of the matched cohort showed that as the albumin level decreased, the risk of POD gradually increased, with a significant association between the two, as detailed in Table 2.

**Figure 3:**
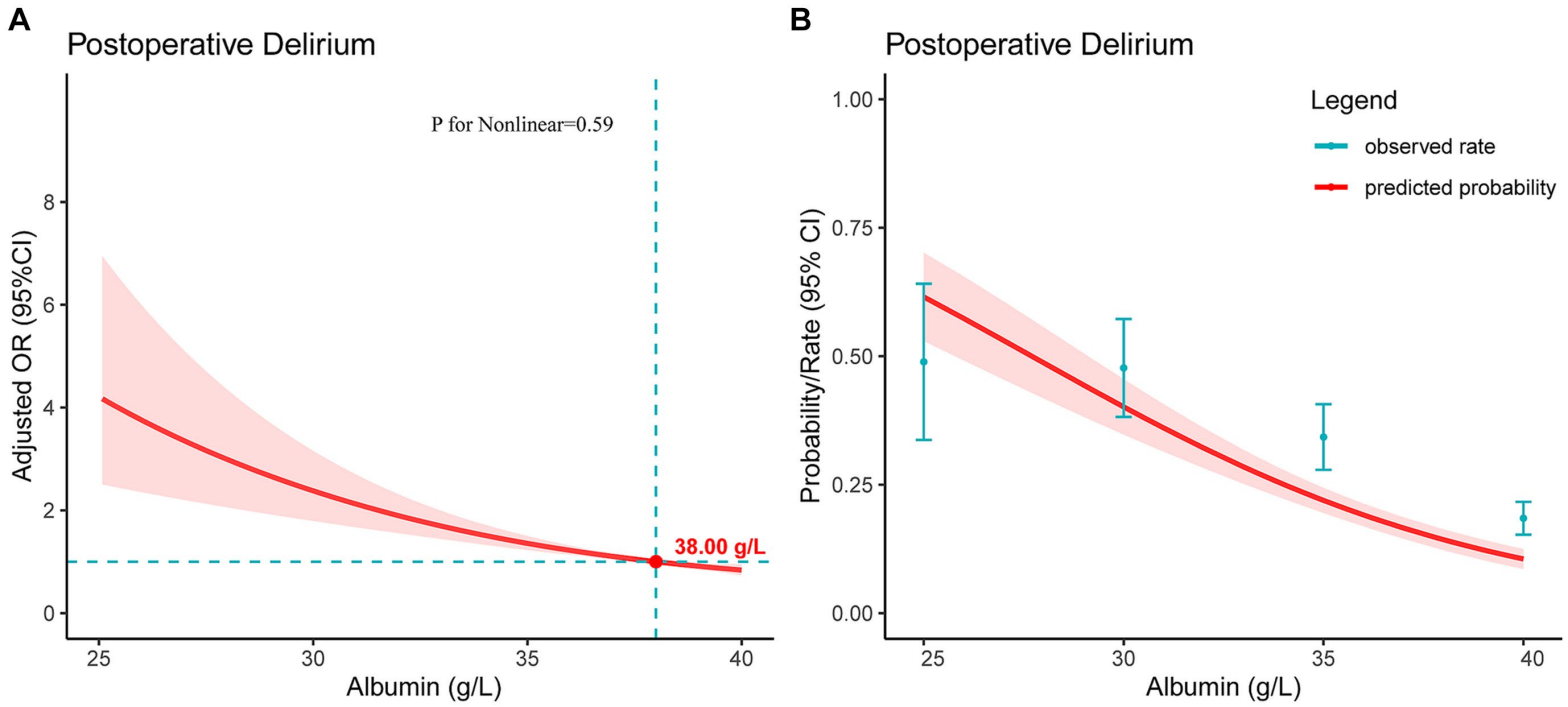
For a line plot depicting the relationship between preoperative serum albumin levels and postoperative delirium in patients with hip fractures. **(A)** The restricted cubic spline plot was used to demonstrate the association between preoperative serum albumin levels and postoperative delirium. The Y-axis represents the adjusted odds ratio, while the X-axis indicates preoperative serum albumin levels. The model has been adjusted for all included covariates. The shaded red area signifies the 95% confidence intervals. The critical value of OR = 1 is denoted by the point. **(B)** The plot illustrates the relationship between preoperative serum albumin levels and the predicted probability of postoperative delirium. The Y-axis represents the predicted probability, while the X-axis indicates preoperative serum albumin levels. The shaded red area signifies the 95% confidence intervals.

Figure 3A presents the restricted cubic spline plot depicting the relationship between preoperative albumin level and POD. The model was adjusted for the clinical severity of albumin and all included covariates. The results indicated a linear dose–response relationship, showing that as the preoperative albumin level decreased, the risk of POD increased (P for Nonlinear = 0.59). Additionally, when the preoperative albumin level fell below the critical value of 38.00 g/L, the risk of POD significantly increased, with an odds ratio of 1. Furthermore, Figure 3B illustrated the association between albumin level and the probability and incidence of POD, highlighting that lower preoperative albumin levels were linked to an increased incidence of POD.

**Figure 4:**
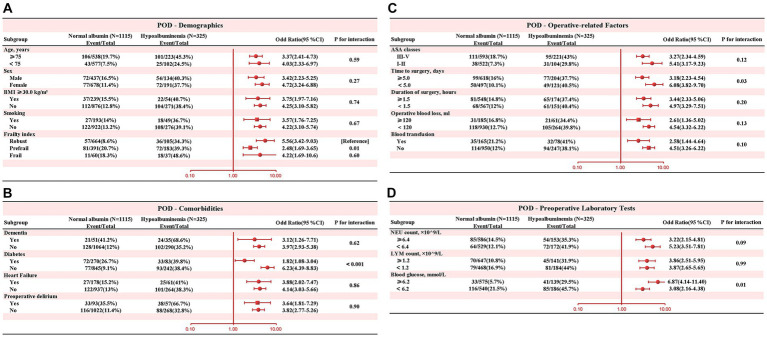
Subgroup analysis was performed to evaluate potential interaction between hypoalbuminemia and each covariate. A value of p less than 0.01 was considered statistically significant. **(A)** Demographics **(B)** Comorbidities **(C)** Operative-related Factors **(D)** Preoperative Laboratory Tests.

In subgroup analysis, Figure 4 depicted the interaction between preoperative albumin level (normal albumin level vs. hypoalbuminemia) and POD, along with other covariates. The results revealed significant interactions between diabetes and hypoalbuminemia (interaction *p* < 0.001). Specifically, patients without diabetes were more prone to develop POD in the presence of preoperative hypoalbuminemia. Therefore, clinicians should be particularly vigilant about POD occurrence when managing patients without diabetes.

## Discussion

POD is a prevalent and significant issue among patients with traumatic hip fractures. While identifying and correcting preoperative risk factors has been a hot topic of concern, current mainstream research (2, 7, 18) has primarily focused on identifying independent risk factors that are difficult to modify or intervene upon, such as advanced age, male gender, dementia, diabetes, ASA classification, and inflammatory indicators such as neutrophil count, lymphocyte count, and C-reactive protein. Recent studies have explored various inflammatory indicators, with Kim et al. (6) suggesting that the C-reactive protein/albumin ratio can predict the occurrence of POD. However, limited research has been conducted on easily modifiable preoperative independent risk factors that are clinically manageable and promotable, such as preoperative serum albumin and blood glucose levels. This research gap hinders clinical practitioners from accurately predicting and intervening in POD caused by preoperative hypoalbuminemia in patients with hip fractures. Therefore, it is crucial to comprehensively understand the varying levels of risk associated with different albumin levels. Doing so will provide valuable insights that can aid clinical doctors in identifying high-risk POD patients early and formulating targeted intervention measures (10).

In our study, through stratified handling of serum albumin levels and multifactorial logistic regression analysis, we obtained precise estimates of the strength of the association between different albumin levels and POD. We found a linear dose–response relationship between serum albumin levels and POD in hip fracture patients: the lower the albumin level, the higher the incidence of POD. Moreover, when the preoperative albumin level was below 38.00 g/L, the risk of POD significantly increased. This finding is consistent with Cabrerizo et al. (19), who also indicated an elevated risk of postoperative complications in geriatric hip fracture patients with albumin levels below 38 g/L. Additionally, subgroup analysis demonstrated a noteworthy interaction between hypoalbuminemia and diabetes in individuals with hip fractures. Nevertheless, cautious interpretation of the interaction effect on diabetes is warranted, considering factors such as sample size. Moreover, the association between preoperative diabetes and preoperative hypoalbuminemia requires further investigation and validation in future studies.

Although the precise mechanism underlying POD remains unknown, various theoretical hypotheses have been proposed in mainstream research to explain its pathogenesis (20–22). These include alterations in central neurotransmitters, decreased brain metabolic levels, inadequate cerebral perfusion, and neuroinflammation. The neuroinflammation hypothesis proposes that external stressors such as surgery and trauma can trigger a systemic immune inflammatory response (6, 23). During a systemic immune inflammatory response, excessive expression of pro-inflammatory cytokines activates vascular endothelial cells and perivascular cells at the blood–brain barrier (6, 24). This disrupts or increases the permeability of the blood–brain barrier, enhancing the transport of peripheral inflammatory cytokines to the brain (9), subsequently activating microglial cells to generate an inflammatory response, interfering with synaptic connections and transmission (7, 25), and even leading to neuronal ischemia and apoptosis (6, 26). In summary, inflammation plays a crucial role in the occurrence of POD.

Albumin is commonly deficient in the older (27, 28). Considering serum albumin levels solely as a nutritional indicator is narrow-minded. Serum albumin not only reflects nutritional status but also closely relates to antioxidant capacity, free radical scavenging, and other functions (15, 29). Albumin is widely distributed in blood, interstitial fluid, and cells, accounting for three-quarters of plasma antioxidant capacity (30, 31). Studies have shown that the antioxidant and free radical scavenging effects of albumin depend on its strong ligand-binding properties, enabling it to bind with free fatty acids, Cu2+, Fe3+, etc., preventing their peroxidation reactions and the formation of reactive oxygen species (30, 32). Additionally, the cysteine at position 34 in albumin contains free thiol, which can scavenge hydroxyl radicals (33).

Taylor et al. (34) conducted a study demonstrating the association between POD and disruption of the blood–brain barrier. They utilized the cerebrospinal fluid/plasma albumin ratio (CPAR) as a reliable measure of blood–brain barrier permeability. Their findings revealed that the severity of delirium was positively correlated with an increase in CPAR, suggesting a link between delirium and blood–brain barrier breakdown. Similarly, Devinney et al. (35) employed a similar methodology and observed that patients who were experiencing delirium exhibited a significantly greater increase in CPAR from preoperative to postoperative 24 h, compared to those who did not experience delirium. Based on these results, they concluded that the heightened permeability of the blood–brain barrier after surgery is independently associated with the occurrence of POD. These changes in blood–brain barrier permeability not only facilitate the transportation of inflammatory factors but also lead to the escape of albumin from the serum into the brain, where it acts as a major extracellular scavenger and antioxidant, perhaps serving as a beneficial mechanism to prevent excessive inflammation and avoid damage to the body (30).

However, in the central nervous system, low serum albumin levels appear to be insufficient in fully exerting their antioxidant effects and capturing free radicals (10, 36, 37). This defense mechanism is hindered in the central nervous system, which can lead to cognitive impairment (10, 38). Additionally, normal serum albumin levels can provide the cell and immune system with essential amino acids and nitrogen-containing substances necessary for body construction, thereby optimizing the composition ratio between albumin, acute phase proteins, and other inflammatory factors to enhance the body’s immune response capabilities (39–43).

Although there is controversy surrounding the benefits of exogenous supplementation of serum albumin, based on existing knowledge, we must consider the impact of hypoalbuminemia on inflammatory activity and the risks it portends (44). This aligns with previous research showing that nutritional supplementation can reduce the occurrence of acute mental disorders during acute trauma and postoperatively and improve clinical outcomes for patients (5, 45–47). It has been widely acknowledged that early identification and intervention in modifiable risk factors are currently the most effective strategy for preventing and managing POD’s complexity (10, 48). Furthermore, assessing preoperative serum albumin levels assists clinicians in identifying high-risk POD patients at an earlier stage. Thus, the objective of our study is to encourage clinicians to emphasize the assessment and management of preoperative serum albumin levels.

### Limitations

There are several limitations that should be acknowledged in our study. Firstly, it is important to recognize that our study design is retrospective. While our findings demonstrate a strong association between exposure and outcomes, it is crucial to note that causality cannot be inferred. Secondly, the management of preoperative serum albumin levels within a relatively short timeframe poses significant challenges in clinical practice, potentially compromising the role of preoperative serum albumin levels as a modifiable risk factor. Thirdly, POD has multifactorial origins and complex mechanisms, making it challenging to address its occurrence solely through targeting a single modifiable risk factor. Therefore, we recommend adopting a comprehensive, multifactorial management approach to effectively mitigate the occurrence of POD. Lastly, it is important to acknowledge that our study was conducted at a single center. Given the limitations in sample size and the homogeneity of the population, generalizing our study findings to all medical centers may be challenging.

## Conclusion

In patients undergoing surgical treatment for hip fractures, we observed a linear dose–response relationship between preoperative serum albumin levels and the incidence of POD. Our findings suggest that maintaining a preoperative serum albumin level above 38 g/L may lead to more favorable outcomes. Consequently, we recommend that clinicians adopt standardized protocols for managing preoperative serum albumin levels, highlighting the significant impact of hypoalbuminemia on inflammatory activity and predictive risk. In order to further investigate the potential benefits, it will be crucial to conduct randomized controlled trials assessing the outcome effects of tightly controlling preoperative serum albumin levels in patients with hip fractures.

## Data availability statement

The raw data supporting the conclusions of this article will be made available by the authors, without undue reservation.

## Ethics statement

The studies involving humans were approved by Ethics Committee of Dandong Central Hospital (Approval no. DDZX-20231101). The studies were conducted in accordance with the local legislation and institutional requirements. The ethics committee/institutional review board waived the requirement of written informed consent for participation from the participants or the participants’ legal guardians/next of kin because This is a retrospective cohort study that did not involve the use of patients’ names, addresses, or any other personally identifiable information.

## Author contributions

WW: Conceptualization, Data curation, Formal analysis, Investigation, Methodology, Software, Writing – original draft, Writing – review & editing. WY: Writing – review & editing. WT: Writing – review & editing. YL: Writing – original draft. QL: Writing – review & editing. WD: Writing – review & editing.
