# Supplementary material for: Association between preoperative albumin levels and postoperative delirium in geriatric hip fracture patients
Source: Front Med (Lausanne). 2024 Feb 14;11:1344904. doi: 10.3389/fmed.2024.1344904 (PMC10899384; doi:10.3389/fmed.2024.1344904)
Supplement: Supplementary file 1 [file Data_Sheet_1.docx]

**Appendix:**

**e-Table1 Studies on Risk Factors (Covariates) Related to Postoperative Delirium**

**e-Table2 List of 27 variables included in the frailty index**

**e-Table3 Multivariate Analysis for Postoperative Delirium**

**e-Table4 Characteristics of patients before and after propensity score matching based on preoperative albumin levels (low < 35 g/L vs. normal ≥ 35 g/L)**

**e-Table1 Studies on Risk Factors (****Covariates) Related to Postoperative Delirium**

| **Covariates** | **Studies** |
| --- | --- |
| Demographics |  |
| Age | Wu[1]; Yang[2]; Kong[3]; Kim[4]; Radinovic[5]; Duppils[6]; Rizk[7]; Guo[8]; Wang[9]; Rui He[10]; Haynes[11]; Ahn[12]; Lee HB[13]; Smith[14] |
| Male | Wu[1]; Qi[15]; Yang[2]; Haynes[11]; Ahn[12]; Lee HB[13] |
| BMI | Wu[1]; Kong[3]; Juliebo[16]; Lee HB[13] |
| Smoking | Wu[1]; Kim[4]; Smith[14] |
| Comorbidities |  |
| Dementia | Wu[1]; Kim[4]; Arshi[17]; Lee KH[18]; Chrispal[19]; Haynes[11]; Smith[14]; Tanaka[20] |
| Diabetes | Qi[15]; Wang[9]; Wang[21]; Rui He[10]; Haynes[11]; Ahn[12] |
| Heart Failure | Wu[1]; Yang[2]; Morrison[22]; Smith[14] |
| Preoperative delirium | Costa-Martins[23]; Qi[15]; Kong[3]; Kim[4]; Arshi[17]; Rizk[7]; Agrawal[24] |
| Operative-related Factors |  |
| ASA | Wu[1]; Kim[4]; Arshi[17]; Wang[9]; Haynes[11]; Smith[14] |
| Time to surgery | Wu[1]; Juliebo[16]; Lee HB[13] |
| Duration of surgery | Kong[3]; Ravi[25]; Guo[8]; Chrispal[19]; Lee HB[13] |
| Operative blood loss | Kong[3]; Shen[26] |
| Blood transfusion | Wu[1]; Zanden[27]; Guo[8] |
| Preoperative Laboratory Tests |  |
| NEU count ×109/L | Rui He[10]; McNeil[28] |
| LYM count ×109/L | Rui He[10]; McNeil[28]; Tanaka[20] |
| Blood glucose mmol/L | Liu[29]; Guo[8] |

**References:**

1. Wu J, Yin Y, Jin M, Li B (2021) The risk factors for postoperative delirium in adult patients after hip fracture surgery: a systematic review and meta-analysis. International journal of geriatric psychiatry 36:3-14. <https://doi.org/10.1002/gps.5408>

2. Yang Y, Zhao X, Dong T, Yang Z, Zhang Q, Zhang Y (2017) Risk factors for postoperative delirium following hip fracture repair in elderly patients: a systematic review and meta-analysis. Aging clinical and experimental research 29:115-126. <https://doi.org/10.1007/s40520-016-0541-6>

3. Kong D, Luo W, Zhu Z, Sun S, Zhu J (2022) Factors associated with post-operative delirium in hip fracture patients: what should we care. European journal of medical research 27:40. <https://doi.org/10.1186/s40001-022-00660-9>

4. Kim EM, Li G, Kim M (2020) Development of a Risk Score to Predict Postoperative Delirium in Patients With Hip Fracture. Anesthesia and analgesia 130:79-86. <https://doi.org/10.1213/ane.0000000000004386>

5. Radinovic K, Markovic-Denic L, Dubljanin-Raspopovic E, Marinkovic J, Milan Z, Bumbasirevic V (2015) Estimating the effect of incident delirium on short-term outcomes in aged hip fracture patients through propensity score analysis. Geriatrics & gerontology international 15:848-855. <https://doi.org/10.1111/ggi.12358>

6. Duppils GS, Wikblad K (2000) Acute confusional states in patients undergoing hip surgery. a prospective observation study. Gerontology 46:36-43. <https://doi.org/10.1159/000022131>

7. Rizk P, Morris W, Oladeji P, Huo M (2016) Review of Postoperative Delirium in Geriatric Patients Undergoing Hip Surgery. Geriatric orthopaedic surgery & rehabilitation 7:100-105. <https://doi.org/10.1177/2151458516641162>

8. Guo Y, Jia P, Zhang J, Wang X, Jiang H, Jiang W (2016) Prevalence and risk factors of postoperative delirium in elderly hip fracture patients. The Journal of international medical research 44:317-327. <https://doi.org/10.1177/0300060515624936>

9. Wang CG, Qin YF, Wan X, Song LC, Li ZJ, Li H (2018) Incidence and risk factors of postoperative delirium in the elderly patients with hip fracture. Journal of orthopaedic surgery and research 13:186. <https://doi.org/10.1186/s13018-018-0897-8>

10. He R, Wang F, Shen H, Zeng Y, LijuanZhang (2020) Association between increased neutrophil-to-lymphocyte ratio and postoperative delirium in elderly patients with total hip arthroplasty for hip fracture. BMC psychiatry 20:496. <https://doi.org/10.1186/s12888-020-02908-2>

11. Haynes MS, Alder KD, Toombs C, Amakiri IC, Rubin LE, Grauer JN (2021) Predictors and Sequelae of Postoperative Delirium in a Geriatric Patient Population With Hip Fracture. Journal of the American Academy of Orthopaedic Surgeons Global research & reviews 5. <https://doi.org/10.5435/JAAOSGlobal-D-20-00221>

12. Ahn EJ, Bang SR (2022) Risk factors associated with treatment of hyperactive postoperative delirium in elderly patients following hip fracture surgery under regional anesthesia: a nationwide population-based study. Brazilian journal of anesthesiology (Elsevier) 72:213-219. <https://doi.org/10.1016/j.bjane.2021.03.020>

13. Lee HB, Mears SC, Rosenberg PB, Leoutsakos JM, Gottschalk A, Sieber FE (2011) Predisposing factors for postoperative delirium after hip fracture repair in individuals with and without dementia. Journal of the American Geriatrics Society 59:2306-2313. <https://doi.org/10.1111/j.1532-5415.2011.03725.x>

14. Smith TO, Cooper A, Peryer G, Griffiths R, Fox C, Cross J (2017) Factors predicting incidence of post-operative delirium in older people following hip fracture surgery: a systematic review and meta-analysis. International journal of geriatric psychiatry 32:386-396. <https://doi.org/10.1002/gps.4655>

15. Qi YM, Li YJ, Zou JH, Qiu XD, Sun J, Rui YF (2022) Risk factors for postoperative delirium in geriatric patients with hip fracture: A systematic review and meta-analysis. Frontiers in aging neuroscience 14:960364. <https://doi.org/10.3389/fnagi.2022.960364>

16. Juliebø V, Bjøro K, Krogseth M, Skovlund E, Ranhoff AH, Wyller TB (2009) Risk factors for preoperative and postoperative delirium in elderly patients with hip fracture. Journal of the American Geriatrics Society 57:1354-1361. <https://doi.org/10.1111/j.1532-5415.2009.02377.x>

17. Arshi A, Lai WC, Chen JB, Bukata SV, Stavrakis AI, Zeegen EN (2018) Predictors and Sequelae of Postoperative Delirium in Geriatric Hip Fracture Patients. Geriatric orthopaedic surgery & rehabilitation 9:2151459318814823. <https://doi.org/10.1177/2151459318814823>

18. Lee KH, Ha YC, Lee YK, Kang H, Koo KH (2011) Frequency, risk factors, and prognosis of prolonged delirium in elderly patients after hip fracture surgery. Clinical orthopaedics and related research 469:2612-2620. <https://doi.org/10.1007/s11999-011-1806-1>

19. Chrispal A, Mathews KP, Surekha V (2010) The clinical profile and association of delirium in geriatric patients with hip fractures in a tertiary care hospital in India. The Journal of the Association of Physicians of India 58:15-19

20. Tanaka T (2016) Factors predicting perioperative delirium and acute exacerbation of behavioral and psychological symptoms of dementia based on admission data in elderly patients with proximal femoral fracture: A retrospective study. Geriatrics & gerontology international 16:821-828. <https://doi.org/10.1111/ggi.12560>

21. Wang Y, Zhao L, Zhang C, An Q, Guo Q, Geng J, Guo Z, Guan Z (2021) Identification of risk factors for postoperative delirium in elderly patients with hip fractures by a risk stratification index model: A retrospective study. Brain and behavior 11:e32420. <https://doi.org/10.1002/brb3.2420>

22. Morrison RS, Magaziner J, Gilbert M, Koval KJ, McLaughlin MA, Orosz G, Strauss E, Siu AL (2003) Relationship between pain and opioid analgesics on the development of delirium following hip fracture. The journals of gerontology Series A, Biological sciences and medical sciences 58:76-81. <https://doi.org/10.1093/gerona/58.1.m76>

23. Costa-Martins I, Carreteiro J, Santos A, Costa-Martins M, Artilheiro V, Duque S, Campos L, Chedas M (2021) Post-operative delirium in older hip fracture patients: a new onset or was it already there? European geriatric medicine 12:777-785. <https://doi.org/10.1007/s41999-021-00456-w>

24. Agrawal S, Turk R, Burton BN, Ingrande J, Gabriel RA (2020) The association of preoperative delirium with postoperative outcomes following hip surgery in the elderly. Journal of clinical anesthesia 60:28-33. <https://doi.org/10.1016/j.jclinane.2019.08.015>

25. Ravi B, Pincus D, Choi S, Jenkinson R, Wasserstein DN, Redelmeier DA (2019) Association of Duration of Surgery With Postoperative Delirium Among Patients Receiving Hip Fracture Repair. JAMA network open 2:e190111. <https://doi.org/10.1001/jamanetworkopen.2019.0111>

26. Shen Y, Shen HL, Zhang W, Fang XT (2013) [Risk factors for delirium of elderly patients undergoing hip fracture operation]. Zhonghua yi xue za zhi 93:3276-3279

27. van der Zanden V, Beishuizen SJ, Swart LM, de Rooij SE, van Munster BC (2017) The Effect of Treatment of Anemia with Blood Transfusion on Delirium: A Systematic Review. Journal of the American Geriatrics Society 65:728-737. <https://doi.org/10.1111/jgs.14564>

28. McNeil JB, Hughes CG, Girard T, Ware LB, Ely EW, Chandrasekhar R, Han JH (2019) Plasma biomarkers of inflammation, coagulation, and brain injury as predictors of delirium duration in older hospitalized patients. PloS one 14:e0226412. <https://doi.org/10.1371/journal.pone.0226412>

29. Liu K, Song Y, Yuan Y, Li Z, Wang X, Zhang W, Li Y, Mi X, Han D, Rong Y, Guo X, Wang G (2022) Type 2 Diabetes Mellitus with Tight Glucose Control and Poor Pre-Injury Stair Climbing Capacity May Predict Postoperative Delirium: A Secondary Analysis. Brain sciences 12. <https://doi.org/10.3390/brainsci12070951>

**e-Table2 List of 27 variables included in the frailty index**

|  | **Definition according to baseline self-report, physical measurements, or both** | **Coding of variables** |
| --- | --- | --- |
| **1** | Self-reported diagnosis of hypertension by a doctor, self-reported use of antihypertension drugs, systolic blood pressure measured to be ≥140 mmHg, or diastolic blood pressure measured to be ≥90 mmHg | Yes=1; No=0 |
| **2** | Self-reported diagnosis of heart disease by a doctor | Yes=1; No=0 |
| **3** | Self-reported diagnosis of stroke or transient ischemic attack by a doctor | Yes=1; No=0 |
| **4** | Self-reported diagnosis of emphysema or chronic bronchitis by a doctor | Yes=1; No=0 |
| **5** | Self-reported diagnosis of tuberculosis by a doctor | Yes=1; No=0 |
| **6** | Self-reported diagnosis of asthma by a doctor | Yes=1; No=0 |
| **7** | Self-reported diagnosis of peptic ulcer by a doctor | Yes=1; No=0 |
| **8** | Self-reported diagnosis of gallstone disease, with or without cholecystitis, by a doctor | Yes=1; No=0 |
| **9** | Self-reported diagnosis of rheumatoid arthritis by a doctor | Yes=1; No=0 |
| **10** | Self-reported diagnosis of fracture by a doctor | Yes=1; No=0 |
| **11** | Self-reported diagnosis of neurasthenia by a doctor | Yes=1; No=0 |
| **12** | Self-reported diagnosis of diabetes, fasting blood glucose measured to be ≥7.0 mmol/L, or random blood glucose measured to be ≥11.1 mmol/L | Yes=1; No=0 |
| **13** | Self-reported diagnosis of cancer by a doctor | Yes=1; No=0 |
| **14** | Self-reported diagnosis of chronic kidney disease by a doctor | Yes=1; No=0 |
| **15** | if you were walking on level ground with other healthy people of the same age, would you usually become short of breath or slow down because of chest discomfort? | Yes=1; No=0 |
| **16** | During the past month, did you have any of the following for ≥3 days per week; (1) taking ≥30 min to fall asleep after going to bed or waking up in the middle of the night; (2) waking up early and not being able to go back to sleep; or (3) having difficulty staying alert while at work eating, or meeting people during the daytime? | Yes=1; No=0 |
| **17** | How often do you have bowel movements each week? | ＜3 times per week=1; Other=0 |
| **18** | During the past 12 months, did you have pain or discomfort in your body lasting ≥3 months that interfered with your life? | Yes=1; No=0 |
| **19** | During the past 12 months, have you developed a frequent cough? | Yes, for ≥3 months=1; Yes, for ＜3 months=0.5; No=0 |
| **20** | Do you brush your teeth rarely or never, or have false teeth? | Yes=1; No=0 |
| **21** | Physical activity in the past 12 months, including the usual type and duration of activities in occupational, commuting, domestic and leisure time-related domains | Lowest quintile stratified by sex=1; Other=0 |
| **22** | During the past 12 months, have you lost weight (≥2.5 Kg) despite not trying to intentionally lose weight? | Yes=1; No=0 |
| **23** | During the past 12 months, did you feel much sadder, or more depressed, than usual for ≥2 weeks? | Yes=1; No=0 |
| **24** | How is your current general health status? | Poor=1; Fair=0.5; Good=0.25; Excellent=0 |
| **25** | Body-mass index (Kg/m^2^) * | ＜18.5 or ＞28=1; ≥24 and ＜28=0.5; ≥18.5 and ＜24=0 |
| **26** | Measured heart rate, beats per min | ＜60 or ＞100=1; ≥60 and＜100=0 |
| **27** | The ratio of forced expiratory volume in 1S to the forced vital capacity measured to be ＜0.7 | Yes=1; No=0 |
| *Body-mass index was calculated by dividing the weight (Kg) of an individual by their height (m^2^) | | |

**e-Table3 Multivariate Analysis for Postoperative Delirium**

| Characteristics | Univariate | | | Collinearity test | Multivariate | | |
| --- | --- | --- | --- | --- | --- | --- | --- |
|  | OR | 95%CI | p-value | VIF | OR | 95%CI | p-value |
| Demographics |  |  |  |  |  |  |  |
| Age, years | 1.10 | 1.08-1.11 | <0.001 | 1.16 | 1.06 | (1.05-1.10) | <0.001 |
| Male | 1.37 | 1.05-1.78 | 0.02 | 1.25 | 1.63 | (1.18-2.26) | 0.003 |
| BMI (≥30.0 kg/m² VS ＜30.0 kg/m²) | 1.09 | 0.79-1.50 | 0.61 | 1.05 | <NA> | <NA> | <NA> |
| Smoking | 0.96 | 0.68-1.37 | 0.83 | 1.23 | <NA> | <NA> | <NA> |
| Frailty index | 2.04 | 1.66-2.50 | <0.001 | 1.11 | 1.43 | (1.08-1.89) | 0.01 |
| Comorbidities |  |  |  |  |  |  |  |
| Dementia | 5.36 | 3.43-8.38 | <0.001 | 1.02 | 3.65 | (2.11-6.31) | <0.001 |
| Diabetes | 2.28 | 1.72-3.02 | <0.001 | 1.06 | 1.27 | (0.85-1.89) | 0.25 |
| Heart Failure | 1.22 | 0.87-1.71 | 0.25 | 1.04 | 0.96 | (0.64-1.43) | 0.83 |
| Preoperative delirium | 4.78 | 3.36-6.82 | <0.001 | 1.08 | 2.32 | (1.52-3.55) | <0.001 |
| Operative-related Factors |  |  |  |  |  |  |  |
| ASA (≥III VS ＜III) | 2.74 | 2.03-3.68 | <0.001 | 1.10 | 1.40 | (0.98-2.02) | 0.06 |
| Time to surgery, days | 1.06 | 1.01-1.12 | 0.01 | 1.07 | 0.99 | (0.93-1.05) | 0.61 |
| Duration of surgery, hours | 1.16 | 1.01-1.35 | 0.04 | 1.35 | 1.15 | (0.92-1.43) | 0.24 |
| Operative blood loss, ml | 1.001 | 1.0007-1.002 | 0.03 | 1.34 | 1.001 | (0.999-1.002) | 0.36 |
| Blood transfusion | 1.81 | 1.32-2.49 | <0.001 | 1.11 | 0.83 | (0.54-1.28) | 0.39 |
| Preoperative Laboratory Tests |  |  |  |  |  |  |  |
| NEU count, ×109/L | 1.05 | 1.01-1.10 | 0.04 | 1.09 | 0.97 | (0.91-1.02) | 0.22 |
| LYM count, ×109/L | 0.59 | 0.46-0.77 | <0.001 | 1.04 | 0.89 | (0.71-1.13) | 0.34 |
| Blood glucose, mmol/L | 1.24 | 1.19-1.30 | <0.001 | 1.09 | 1.23 | (1.16-1.30) | <0.001 |
| Albumin, g/L | 0.87 | 0.84-0.89 | <0.001 | 1.15 | 0.90 | (0.87-0.93) | <0.001 |

NA, Not Applicable; CI, Confidence Interval; OR, Odds Ratio; BMI, Body Mass Index; ASA, American Society of Anesthesiologists; NEU, Neutrophils; LYM, Lymphocytes; VIF, Variance Inflation Factor.

**e-Table4 Characteristics of patients before and after propensity score matching based on preoperative albumin levels (low < 35 g/L vs. normal ≥ 35 g/L)**

| Characteristics | Before matching | | | After matching | | |
| --- | --- | --- | --- | --- | --- | --- |
|  | Normal albumin (n=1115) | Hypoalbuminemia (n=325) | SMD | Normal albumin (n=305) | Hypoalbuminemia (n=305) | SMD |
| Demographics |  | | | | | |
| Age, years (Median, IQR) | 74 (16) | 80 (15) | 0.55 | 79 (14) | 79 (15) | 0.01 |
| Male (n, %) | 437(39.2%) | 134(41.2%) | 0.04 | 118(38.7%) | 127(41.6%) | 0.06 |
| BMI ≥30.0 kg/m² (n, %) | 239(21.4%) | 54(16.6%) | 0.12 | 41(13.4%) | 48(15.7%) | 0.06 |
| Smoking (n, %) | 193(17.3%) | 49(15.1%) | 0.06 | 45(14.8%) | 46(15.1%) | 0.009 |
| Frailty index |  | | | | | |
| Robust (n, %) | 664(59.6%) | 105(32.3%) | 0.54 | 108(35.4%) | 104(34.1%) | 0.01 |
| Prefrail (n, %) | 391(35.1%) | 183(56.3%) |  | 165(54.1%) | 171(56.1%) |  |
| Frail (n, %) | 60(5.4%) | 37(11.4%) |  | 32(10.5%) | 30(9.8%) |  |
| Comorbidities |  | | | | | |
| Dementia (n, %) | 51(4.6%) | 35(10.8%) | 0.23 | 25(8.2%) | 21(6.9%) | 0.05 |
| Diabetes (n, %) | 270(24.2%) | 83(25.5%) | 0.03 | 73(23.9%) | 77(25.2%) | 0.03 |
| Heart Failure (n, %) | 178(16.0%) | 61(18.8%) | 0.07 | 66(21.6%) | 58(19%) | 0.07 |
| Preoperative delirium (n, %) | 93(8.3%) | 57(17.5%) | 0.28 | 50(16.4%) | 47(15.4%) | 0.03 |
| Operative-related Factors |  | | | | | |
| ASA classes ≥III (n, %) | 593(53.2%) | 221(68.0%) | 0.31 | 197(64.6%) | 203(66.6%) | 0.04 |
| Time to surgery, days (Median, IQR) | 5 (4) | 5 (4) | 0.18 | 5 (4) | 5 (3) | 0.007 |
| Duration of surgery, hours (Median, IQR) | 1.43 (0.75) | 1.50 (0.83) | 0.09 | 1.5 (0.88) | 1.5 (0.83) | 0.05 |
| Operative blood loss, ml (Median, IQR) | 120 (116) | 136 (100) | 0.10 | 130 (107) | 135 (100) | 0.01 |
| Blood transfusion (n, %) | 165(14.8%) | 78(24.0%) | 0.23 | 66(21.6%) | 69(22.6%) | 0.02 |
| Preoperative Laboratory Tests |  | | | | | |
| NEU count, ×10^9^/L (Median, IQR) | 6.50 (3.40) | 6.20 (3.00) | 0.03 | 6.3 (3.1) | 6.2 (3.25) | 0.003 |
| LYM count, ×10^9^/L (Median, IQR) | 1.23 (0.74) | 1.10 (0.50) | 0.17 | 1.1 (0.73) | 1.1 (0.5) | 0.01 |
| Blood glucose, mmol/L (Median, IQR) | 6.10 (2.10) | 6.50 (2.30) | 0.01 | 6.3 (2.35) | 6.4 (2.3) | 0.005 |

SMD, Standardized Mean Difference; CI, Confidence Interval; OR, Odds Ratio; BMI, Body Mass Index; ASA, American Society of Anesthesiologists; NEU, Neutrophils; LYM, Lymphocytes.
